# Supplementary material for: Cloning and comparative modeling identifies a highly stress tolerant Cu/Zn cytosolic super oxide dismutase 2 from a drought tolerant maize inbred line
Source: PeerJ. 2023 Mar 13;11:e14845. doi: 10.7717/peerj.14845 (PMC10019340; doi:10.7717/peerj.14845)
Supplement: Supplemental Information 2 [file peerj-11-14845-s002.pdf]

Cloning and comparative modeling identifies a highly stress tolerant Cu/Zn cytosolic Super Oxide Dismutase 2 from a drought tolerant maize inbred line

Anuradha Gautam[1], Fatima Nazish Khan[2,3], Surabhi Priya[1,] Krishan Kumar[1], Shivani Sharda[4], Tanushri Kaul[5], Ishwar Singh[1], Sapna Langyan[6]*, Pranjal Yadava[1,2]*

[1] ICAR- Indian Institute of Maize Research, Pusa Campus, New Delhi, 110012, India

[2] Division of Plant Physiology, Indian Agricultural Research Institute, Pusa, New Delhi, 110012, India

[3] Department of Biotechnology, Jamia Millia Islamia, Jamia Nagar, New Delhi, 110025, India

[4] Amity Institute of Biotechnology. Amity University, Sector-125, Noida, UP, 201313, India

[5] International Centre for Genetic Engineering and Biotechnology, Aruna Asaf Ali Marg, New Delhi, 110067, India

[6] Division of Germplasm Evaluation, ICAR- National Bureau of Plant Genetic Resources, Pusa Campus, New Delhi, India

SUPPLEMENTARY FIGURES

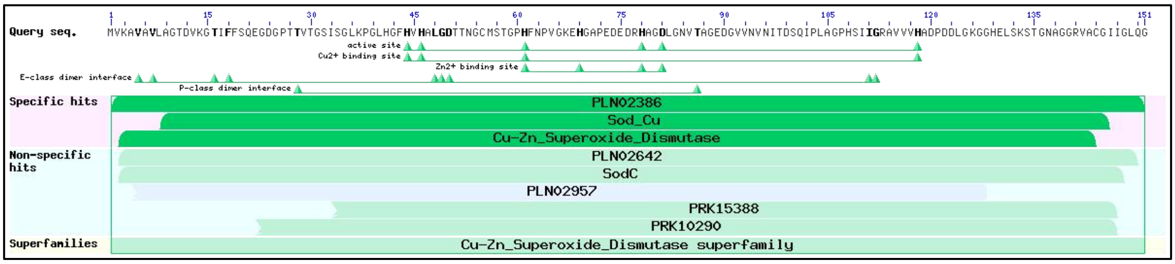

Figure S1: Conserved domains in the cloned ZmSOD2 protein from drought tolerant inbred HKI 335 (ALF00121.1).

```
1st line -> residue numeration
2nd line -> query amino acid sequence
3rd line -> trans-membrane domain prediction (T-TM region, N-soluble part)

>  1                                                            60
   MVKAVAVLAGTDVKGTIFFSQEGDGPTTVTGSISGLKPGLHGFHVHALGDTTNGCMSTGP
   NNNNNNNNNNNNNNNNNNNNNNNNNNNNNNNNNNNNNNNNNNNNNNNNNNNNNNNNNNNN

>  61                                                           120
   HFNPVGKEHGAPEDEDRHAGDLGNVTAGEDGVVNVNITDSQIPLAGPHSIIGRAVVVHAD
   NNNNNNNNNNNNNNNNNNNNNNNNNNNNNNNNNNNNNNNNNNNNNNNNNNNNNNNNNNNN

> 121                           151
   PDDLGKGGHELSKSTGNAGGRVACGIIGLQG
   NNNNNNNNNNNNNNNNNNNNNNNNNNNNNNN

END_SECTION
```

Figure S2: Trans-membrane domain prediction showing that protein is soluble and no trans-membrane region is found.
